# Maternal coronary heart disease and mortality following hypertensive disorders of pregnancy and/or diabetes

**DOI:** 10.1186/s12933-025-02811-8

**Published:** 2025-07-11

**Authors:** Angela M. Malek, Dulaney A. Wilson, Julio Mateus, Emily A. Ash, Tanya N. Turan, Daniel T. Lackland, Kelly J. Hunt

**Affiliations:** 1 Department of Public Health Sciences, Medical University of South Carolina, Charleston, SC 29425 USA; 2 Department of Obstetrics & Gynecology, Maternal-Fetal Medicine Division, Atrium Health, Charlotte, NC 28204 USA; 3 Department of Neurology, Medical University of South Carolina, Charleston, SC 29425 USA

**Keywords:** Diabetes, Chronic hypertension, Coronary heart disease, Mortality, Hypertensive disorders of pregnancy

## Abstract

**Background:**

Pre-pregnancy hypertension (HTN), hypertensive disorders of pregnancy (HDP), and diabetes have been linked to increased risk of post-pregnancy coronary heart disease (CHD) and all-cause mortality, but few studies have investigated their cumulative impact. This study aimed to assess the potential relationship between pre-pregnancy HTN, HDP, and diabetes and their cumulative impact on maternal cardiovascular outcomes defined as incident CHD and all-cause mortality within 5 years of delivery and over the entire study period (up to 14 years after delivery).

**Methods:**

This retrospective cohort study included 430,545 women aged 12–49 with ≥ 1 singleton, live birth in South Carolina (2004–2016) including non-Hispanic White (NHW; 59.2%), non-Hispanic Black (NHB; 31.4%), and Hispanic (9.4%) women. Birth certificate and hospitalization/emergency department (ED) visit data defined pre-pregnancy HTN, HDP (preeclampsia, eclampsia, gestational HTN), and diabetes (pre-pregnancy, gestational). Hospitalization/ED visit and death certificate data defined incident CHD and all-cause mortality. Covariate-adjusted Cox proportional hazard models were used to assess associations between CHD and mortality by exposure.

**Results:**

After adjustment for covariates relative to women without any of the three conditions (diabetes, pre-pregnancy HTN, HDP), incident CHD risk was increased within 5 years of delivery for women with diabetes (HR = 1.57; 95% CI 1.28–1.92), HDP (HR = 1.85; 95% CI 1.60–2.15), HDP and diabetes (HR = 2.29; 95% CI 1.73–3.03), HDP and pre-pregnancy HTN (HR = 3.13; 95% CI 2.66–3.68), and all three conditions (HR = 4.87; 95% CI 3.95–6.01). All-cause mortality risk was increased for diabetes (HR = 1.34; 95% CI 1.01–1.78), HDP and pre-pregnancy HTN (HR = 1.53; 95% CI 1.15–2.03), and all three conditions (HR = 2.25; 95% CI 1.51–3.36), but not HDP or HDP and diabetes.

**Conclusions:**

Within 5 years of delivery, incident CHD and all-cause mortality rates were highest for women with two or three conditions, specifically HDP, diabetes, and/or pre-pregnancy HTN, with all rates higher for NHB than NHW women. Thus, it is critical to implement clinical prevention strategies to improve risk factor screening and identification among women of child-bearing age.

**Supplementary Information:**

The online version contains supplementary material available at 10.1186/s12933-025-02811-8.

## Research Insights (< 200 words)

**What is currently known about this subject? (max. of 3 highlights)**

Pregnant women with pre-pregnancy hypertension (HTN), hypertensive disorders of pregnancy (HDP), or diabetes are at increased risk for post-pregnancy cardiovascular outcomes and mortality.

Many risk factors are shared between pre-pregnancy HTN, HDP, and diabetes.

**What is the key research question? (formatted as a question)**

What is the cumulative impact of pre-pregnancy HTN, HDP, and/or diabetes on post-pregnancy incident coronary heart disease (CHD) and all-cause mortality ≤ 5 years of delivery and the study period (≤ 14 years)?

**What is new? (max. 3 highlights)**

Incident CHD and all-cause mortality rates were highest for women with 2–3 conditions (HDP and diabetes; HDP and pre-pregnancy HTN; HDP, diabetes, and pre-pregnancy HTN). Within categories, rates were higher among non-Hispanic Black than non-Hispanic White women.

Within 5 years of delivery, incident CHD risk was increased among women with diabetes 57%, HDP 85%, HDP and diabetes 2.29-fold, HDP and pre-pregnancy HTN 3.13-fold, and all three conditions 4.87-fold compared to no condition.

All-cause mortality risk was increased for women with diabetes 34%, HDP and pre-pregnancy HTN 53%, and all three conditions 2.25-fold, but not associated with HDP, or HDP and diabetes.

**How might this study influence clinical practice? (max. 1 highlight)**

Our findings highlight the importance of implementing clinical practice strategies among women of child-bearing age that could include patient education, cardiovascular risk factor modification, early identification, and adequate post-partum follow-up care.

## Background

Cardiovascular-related diseases are consistent leading causes of death in the United States (U.S.) [1]. Among pregnant women, pre-pregnancy hypertension (HTN), hypertensive disorders of pregnancy (HDP), and diabetes have all been linked to an increased risk of post-pregnancy cardiovascular outcomes and mortality [2–8]. HDP include gestational HTN, preeclampsia, and eclampsia and affect up to 10% of pregnancies [9, 10]. Many studies have reported associations between pre-pregnancy HTN and HDP with increased short- and long-term morbidity and mortality for the mother and the baby when compared to mothers without these conditions [10–12]. Adverse maternal outcomes include an increased risk of maternal mortality, cardiovascular disease (CVD), myocardial infarction, and coronary heart disease (CHD) [11–13]. Gestational diabetes (GDM) has also been related to CVD [8]. In a European genome-wide genetic association study, relationships were reported between genetically predicted HDP as a composite, gestational hypertension, and preeclampsia/eclampsia and CHD [14]. Furthermore, cardiometabolic factors (diabetes and systolic blood pressure) were observed to partially mediate the association of HDP but not gestational hypertension with coronary artery disease, with mediation analysis not possible for preeclampsia/eclampsia [14]. 

Pre-pregnancy HTN, HDP, and diabetes share many risk factors, with each condition found to be associated with the other [6, 7, 15]. However, less is known regarding the cumulative impact of all three conditions post-pregnancy in terms of maternal CVD outcomes and mortality. While atherosclerosis and endothelial inflammation may be accelerated due to HDP and GDM, it is unknown if HDP and GDM have a synergistic effect on subsequent cardiometabolic disease [16]. 

Maternal race and ethnicity are also associated with post-pregnancy maternal morbidity and mortality [17]. From 2007 to 2018, non-Hispanic White (NHW), non-Hispanic Black (NHB), and Hispanic women experienced increased rates of diabetes (including both during and prior to pregnancy), chronic HTN, and HDP in the U.S. based on the Centers for Disease Control and Prevention (CDC) Natality data [17]. Of these groups, Hispanic and then NHB women had the greatest increases in diabetes, chronic HTN, and HDP [17]. NHB women continued to have the highest prevalence of HDP and chronic HTN with Hispanic women continuing to have the highest prevalence of diabetes [17]. Among U.S. Hispanic/Latina women, age-standardized rates of GDM were also observed to increase significantly from 2011 to 2019, with the rate higher for NHB than NHW women in 2019 (66.6 per 1000 live births; rate ratio = 1.15; 95% CI 1.13–1.18) according to CDC National Center for Health Statistics (NCHS) data [18]. However, some studies investigating the associations of pre-pregnancy or chronic HTN, HDP, and/or diabetes have failed to adequately assess racial/ethnic differences, most often due to limited data collection and reduced power.

Our study aimed to assess the potential relationship between pre-pregnancy HTN, HDP, and diabetes and their cumulative impact on post-pregnancy maternal cardiovascular outcomes defined as incident CHD and all-cause mortality within 5 years of delivery and over the entire study period (up to 14 years after delivery) in a high-risk region in the U.S. Additionally, racial/ethnic differences were assessed as well as an interaction with pre-pregnancy HTN, HDP, and diabetes. Our data is uniquely positioned to assess racial/ethnic differences as this study was conducted in South Carolina (SC), a state in the southeastern region of the U.S. where over one-third of the population identify as non-White [19]. 

## Methods

### Study design and population

**Fig. 1:**
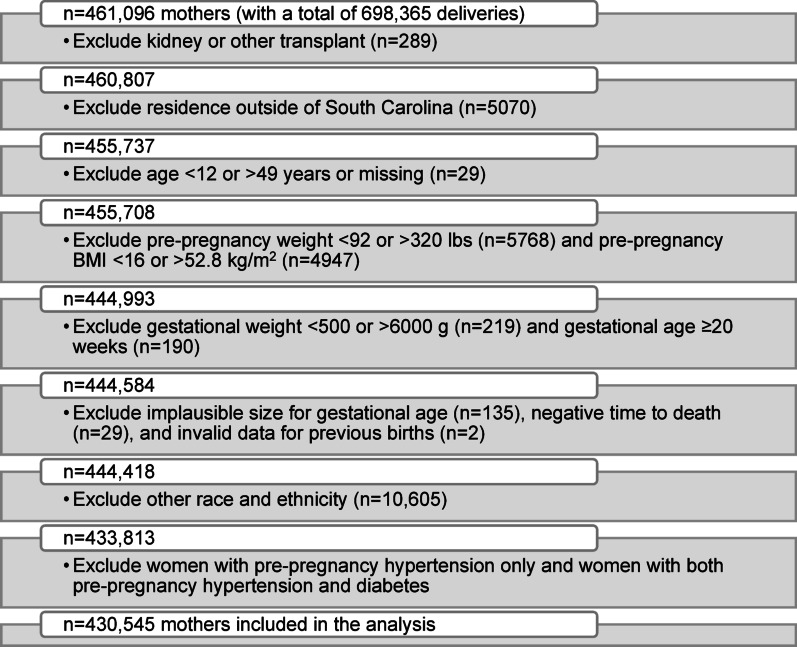
Study flow chart

This retrospective cohort study focused on mothers from the state of SC. These women were between the ages of 12–49 and had ≥ 1 singleton, live birth between 2004 and 2016. The original data set was derived from birth and death certificates from the SC Department of Health and Environmental Control, and hospitalization records and emergency department (ED) visits from the SC Revenue and Fiscal Affairs Office, Health and Demographics Section [2]. Women who had pre-pregnancy HTN alone and pre-pregnancy HTN with diabetes were excluded from the analysis due to low counts. The inclusion and exclusion criteria used for this study are presented in Fig. 1.

## Definitions

Table 1 presents covariates considered for analysis by exposure group. Sociodemographic characteristics considered included: maternal age, race/ethnicity, education, rural/urban, income, primary payer during pregnancy, and receipt of the Special Supplemental Nutrition Program for Women, Infants, and Children (WIC) during pregnancy. Maternal age, race/ethnicity, education level, primary payer, and WIC were available from the birth certificate. WIC is a U.S. federal nutrition program based on income eligibility (between 100% and 185% of the federal poverty income guidelines) and includes those who receive any of the following federal programs: Medicaid, Supplemental Nutrition Assistance Program (SNAP), or Temporary Assistance for Needy Families (TANF) [20]. Race/ethnicity was based on self-report data. For the purpose of this study, women were categorized as NHW (reference group), NHB, and Hispanic race/ethnicity. Women of other race/ethnic groups (*n* = 10,605) were excluded from the analysis due to low counts. Education level was reported as less than high school, high school graduate, some college, and college graduate and above (reference group). Primary payer during pregnancy was classified as Medicaid eligible, private insurance (reference group), self-pay, or other. Rural/urban status was based on Rural-Urban Commuting Area (RUCA) codes by zip code. Median household income per year by U.S. Census zip code of residence was classified by as <$36,000 (reference group); $36,000 to <$54,000; and ≥$54,000 a year per household.

**Table 1:** Characteristics of mothers at index birth in South Carolina overall and by exposure status, 2004–2016

Characteristic, *n* (%) or mean ± SD	Total *n* = 430,545	None *n* = 334,276 (77.6)	Diabetes *n* = 22,914 (5.3)	HDP *n* = 44,491 (10.3)	HDP & Diabetes *n* = 7,055 (1.6)	HDP & Pre-preg HTN *n* = 16,708 (3.9)	HDP, Diabetes, & Pre-preg HTN *n* = 5,101 (1.2)	*p*-value
Maternal age	26.3 ± 6.1	25.9 ± 6.0	29.1 ± 6.1	25.9 ± 6.0	28.6 ± 6.2	28.7 ± 6.3	31.2 ± 6.1	< 0.0001
Race/ethnicity								
NHW	254,897 (59.2)	200,662 (60.0)	13,607 (59.4)	26,501 (59.6)	4,030 (57.1)	7,831 (46.9)	2,266 (44.4)	< 0.0001
NHB	135,311 (31.4)	100,011 (29.9)	6,612 (28.9)	15,340 (34.5)	2,436 (34.5)	8,314 (49.8)	2,598 (50.9)	
Hispanic	40,337 (9.4)	33,603 (10.1)	2,695 (11.8)	2,650 (6.0)	589 (8.3)	563 (3.4)	237 (4.6)	
Education								
< HS	86,299 (20.0)	69,796 (20.9)	3,741 (16.3)	8,404 (18.9)	1,170 (16.6)	2,504 (15.0)	684 (13.4)	< 0.0001
HS graduate	105,844 (24.6)	81,452 (24.4)	5,365 (23.4)	11,365 (25.5)	1,748 (24.8)	4,584 (27.4)	1,330 (26.1)	
Some college	106,371 (24.7)	80,863 (24.2)	5,945 (25.9)	11,563 (26.0)	1,973 (28.0)	4,512 (27.0)	1,515 (29.7)	
≥College graduate	132,031 (30.7)	102,165 (30.6)	7,863 (34.3)	13,159 (29.6)	2,164 (30.7)	5,108 (30.6)	1,572 (30.8)	
Rural ^a^	113,897 (26.5)	88,395 (26.4)	6,072 (26.5)	11,492 (25.8)	1,764 (25.0)	4,771 (28.6)	1,403 (27.5)	0.02
Income ^b^								
Missing	12,392 (2.9)	10,535 (3.2)	573 (2.5)	887 (2.0)	140 (2.0)	183 (1.1)	74 (1.5)	< 0.0001
Under $36K	118,509 (28.3)	90,414 (27.9)	6,000 (26.9)	12,681 (29.1)	2,030 (29.4)	5,698 (34.5)	1,686 (33.5)	
$36K to <$54K	199,700 (47.8)	154,550 (47.7)	10,573 (47.3)	21,283 (48.8)	3,303 (47.8)	7,635 (46.2)	2,356 (46.9)	
≥$54K	99,944 (23.9)	78,777 (24.3)	5,768 (25.8)	9,640 (22.1)	1,582 (22.9)	3,192 (19.3)	985 (19.6)	
Medicaid ^c^	210,000 (49.2)	162,587 (49.1)	9,876 (43.6)	22,841 (51.7)	3,430 (49.0)	8,729 (52.7)	2,537 (50.1)	< 0.0001
WIC	228,970 (54.0)	177,227 (53.9)	11,604 (51.4)	23,867 (54.5)	3,840 (55.1)	9,426 (57.2)	3,006 (59.8)	< 0.0001
Smoking during pregnancy	50,738 (11.8)	39,796 (11.9)	2,715 (11.9)	4,889 (11.0)	831 (11.8)	1,962 (11.8)	545 (10.7)	< 0.0001
Smoking pre-pregnancy	65,854 (15.3)	51,318 (15.4)	3,560 (15.6)	6,680 (15.0)	1,092 (15.5)	2,486 (14.9)	718 (14.1)	0.02
Pre-pregnancy BMI (kg/m^2^**)**	27.0 ± 6.7	26.0 ± 6.1	29.5 ± 7.1	28.8 ± 7.1	32.0 ± 7.5	32.1 ± 7.8	35.6 ± 7.6	< 0.0001
Number of pregnancies prior to index	0.7 ± 1.0	0.7 ± 1.0	0.9 ± 1.1	0.6 ± 1.0	0.8 ± 1.2	0.9 ± 1.2	1.0 ± 1.2	< 0.0001
Incident CHD ^d^	3,859 (0.9)	2,138 (0.6)	295 (1.3)	529 (1.2)	156 (2.2)	477 (2.9)	264 (5.2)	< 0.0001
All-cause mortality ^d^	1,959 (0.5)	1,383 (0.4)	120 (0.5)	216 (0.5)	50 (0.7)	134 (0.8)	56 (1.1)	< 0.0001

CHD, coronary heart disease; HDP, hypertensive disorders of pregnancy; HTN, hypertension; K, thousand; NHB, non-Hispanic Black; NHW, non-Hispanic White; preg, pregnancy; SD, standard deviation; WIC, Special Supplemental Nutrition Program for Women, Infants and Children

^a^ Urban/rural is based on rural-urban commuting area (RUCA) codes by zip code of residence

^b^ Annual household income

^c^ Variables with < 2% missing included: Primary payer during pregnancy (includes Medicaid eligibility, *n* = 3,941), WIC receipt during pregnancy (*n* = 6,652), Smoking during pregnancy (*n* = 192), Smoking pre-pregnancy (*n* = 316)

^d^ The numbers for incident CHD and all-cause mortality are for the entire study period (up to 14 years after delivery)

Behavioral characteristics included smoking during and pre-pregnancy, which were available from the birth certificate. Clinical characteristics included pre-pregnancy body mass index (BMI) and the number of births prior to the index birth, which were available from the birth certificate. For the purpose of this analysis, we modeled BMI as a continuous variable, and a response of “No” was treated as the reference group for all dichotomous variables. Post-pregnancy BMI was not considered as water retention is a common feature of preeclampsia [21]. 

The index pregnancy was the first pregnancy recorded in the dataset for unexposed women and the first recorded pregnancy with either pre-pregnancy HTN or HDP for exposed women. The six mutually exclusive exposure groups consisted of women: (1) without pre-pregnancy HTN, HDP, or diabetes; (2) with diabetes; (3) with HDP; (4) with HDP and diabetes; (5) with HDP and pre-pregnancy HTN; and (6) with HDP, diabetes, and pre-pregnancy HTN. Diagnosis of pre-pregnancy HTN, HDP, and diabetes (pre-pregnancy and gestational) was based on birth certificate information and/or International Classification of Diseases, Ninth and Tenth Revision, Clinical Modification (ICD-9-CM and ICD-10-CM) codes derived from hospitalization/ED visit encounter data (Supplemental Table 1). HDP was defined as gestational HTN, preeclampsia, or eclampsia and was indicated on the birth certificate by a checkbox for gestational HTN. The birth certificate also included checkboxes for pre-pregnancy HTN, pre-pregnancy diabetes, and gestational diabetes. If a mother had a diagnosis of pre-pregnancy or gestational diabetes on the birth certificate, she was considered to have diabetes.

The two outcomes of interest, incident CHD and all-cause mortality within 5 years of delivery and over the entire study period (up to 14 years), were examined between 2005 and 2016 with follow-up through 2017 to allow at least one year of follow-up time after the delivery. Incident CHD was defined by ICD-9/10-CM codes determined through hospitalization and ED visit records as well as ICD-10 codes based on death certificate records. All-cause mortality was determined using death certificates. Displayed in Supplemental Table 1 are the ICD-9/10-CM codes and definitions for the outcomes of interest. The underlying causes of death are shown in Supplemental Table 2.

### Statistical analysis

Statistical analyses were performed using SAS 9.4 [22] and Stata 16 [23]. A statistically significant p-value of 0.05 (95% confidence interval [CI]) was used. Continuous variables were assessed with analysis of variance (ANOVA) tests and reported as means ± standard deviations (SD). Categorical variables were assessed with chi-square tests and reported as frequencies (percentages).

Univariate associations were explored for the exposures and each characteristic in relation to incident CHD and all-cause mortality. Incident CHD and all-cause mortality event rates (per 1,000 person-years) within 5 years and within 14 years of delivery are presented for exposure categories in the cohort overall and by race/ethnicity for NHW and NHB women but not Hispanic women due to low counts (available in Supplemental Table 3). Kaplan-Meier curves for maternal incident CHD and all-cause mortality within 5 years of delivery are presented with the y-axis truncated in the cohort overall and by race/ethnicity for NHW and NHB women. Cox proportional hazard models were used to develop hazard ratios (HRs) and corresponding 95% CIs. Multivariate analyses were run with the total cohort with an interaction between exposure and race/ethnic group included for both outcomes for which the p-values were not significant at 0.87 and 0.94 for CHD and 0.51 and 0.42 for all-cause mortality within 5 years of delivery and the entire study period, respectively, in the fully adjusted models. Because the p-values for an interaction between exposure and race/ethnic group were not significant, regression results are presented for the total cohort. To address missingness, models were run with complete observations. As consistent with the literature, models adjusted for sociodemographic, behavioral, and clinical characteristics. While urban/rural residence was not statistically significant in relation to incident CHD, it was controlled for in the models for consistency with all-cause mortality and the literature. Proportionality was tested with Schoenfeld residuals; all assumptions were met.

## Results

A total of 461,966 women had a live singleton birth between 2004 and 2016 in SC. Of those, 430,545 were eligible for this investigation (59.2% NHW; 31.4% NHB, and 9.4% Hispanic) and were classified among the six exposure groups (see Table 1). These mutually exclusive groups consisted of women: (1) without pre-pregnancy HTN, HDP, or diabetes (77.6%); and with (2) diabetes (5.3%); (3) HDP (10.3%); (4) HDP and diabetes (1.6%); (5) HDP and pre-pregnancy HTN (3.9%); and (6) all three conditions (HDP, pre-pregnancy HTN, and diabetes; 1.2%). A total of 3,859 incident CHD events were recorded over the course of the up-to 14-year study (1,610 within 5 years of delivery), as well as 1,959 deaths (850 within 5 years of delivery).

Table 1 presents characteristics for the cohort overall and by exposure group. Compared to no conditions, women with diabetes regardless of HDP, with HDP and pre-pregnancy HTN, and with all three conditions were more likely to be older, NHB, have lower income, be eligible for Medicaid (except for those with HDP and diabetes), receive WIC during pregnancy, and have more pregnancies before the index birth (except for those with HDP). Women of all exposure groups were more likely to have a higher pre-pregnancy BMI than those with no conditions.

### Pre-pregnancy HTN, HDP, and/or diabetes with incident CHD within 5 years and up to 14 years (entire study period) after delivery

**Fig. 2:**
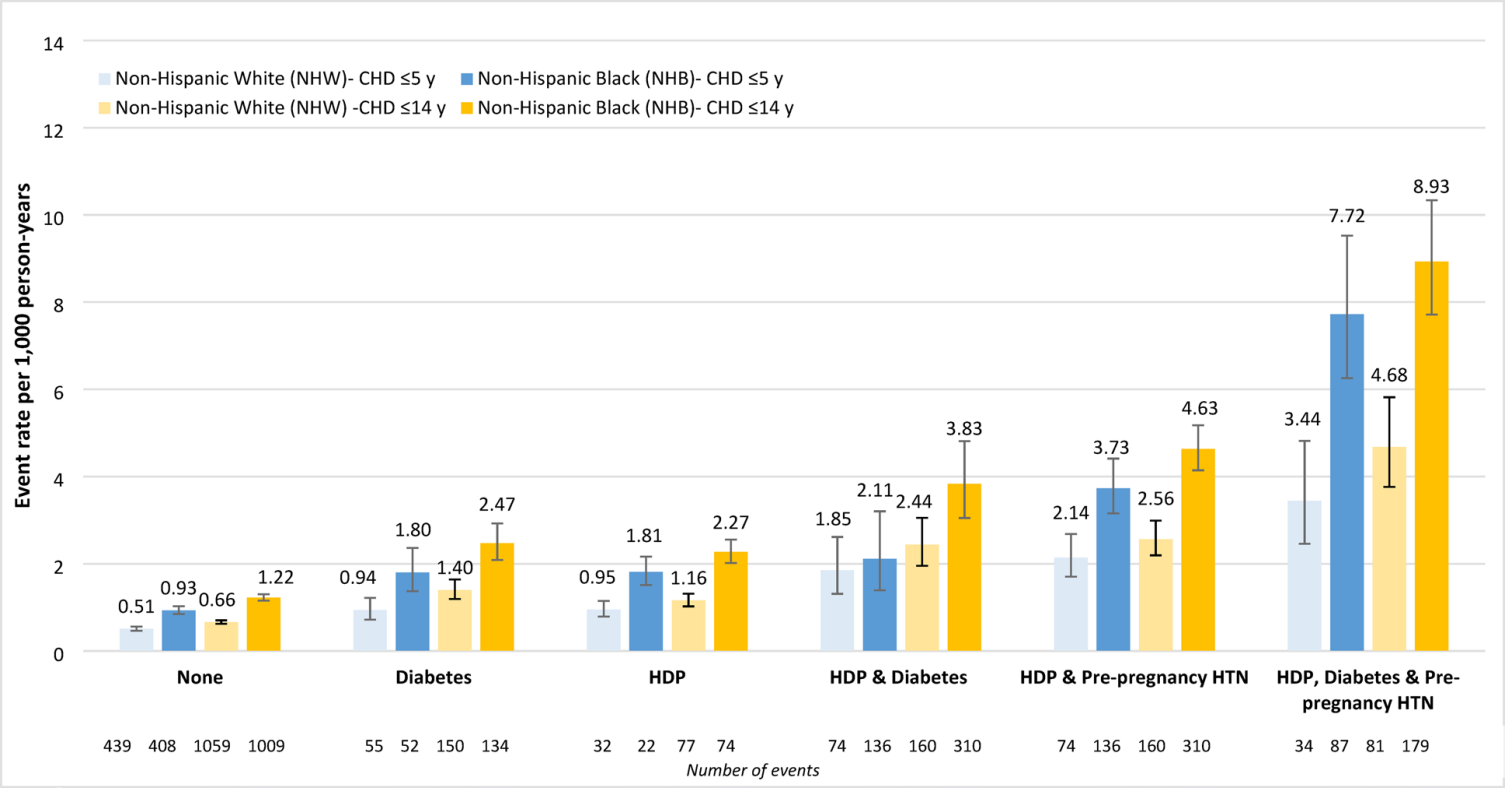
Incident CHD events (per 1,000 person-years) among women ≤ 5 years following delivery and over the entire study period (≤ 14 years) by race/ethnicity and exposure group ^a^. CHD, coronary heart disease; CI, confidence interval; HDP, hypertensive disorders of pregnancy; HR, hazard ratio; HTN, hypertension (pre-pregnancy); NHB, non-Hispanic Black; NHW, non-Hispanic White. ^a^ The total population included Hispanic women; however, the number of events were too low to present race/ethnic-specific events for Hispanic women

Incident CHD event rates (per 1,000 person-years) within 5 years of delivery were highest for women with all three conditions (5.55, *n* = 123), followed by HDP and pre-pregnancy HTN (2.87, *n* = 211), HDP and diabetes (1.88, *n* = 57), HDP (1.22, *n* = 235), diabetes (1.11, *n* = 110), and no exposure (0.61, *n* = 874) (Supplemental Table 3). When examined by race/ethnic group for NHW and NHB women, incident CHD event rates within 5 years of delivery were highest for NHB women in all exposure groups (Fig. 2).

Incident CHD event rates (per 1,000 person-years) within 14 years of delivery were also highest for women with all three conditions (6.75, *n* = 264), followed by HDP and pre-pregnancy HTN (3.65, *n* = 477), HDP and diabetes (2.81, *n* = 156), HDP (1.50, *n* = 529), diabetes (1.62, *n* = 295), and no exposure (0.79, *n* = 2138) (Supplemental Table 3). NHB women similarly experienced the highest CHD rates in all exposure groups within 14 years of delivery (Fig. 2).

**Fig. 3:**
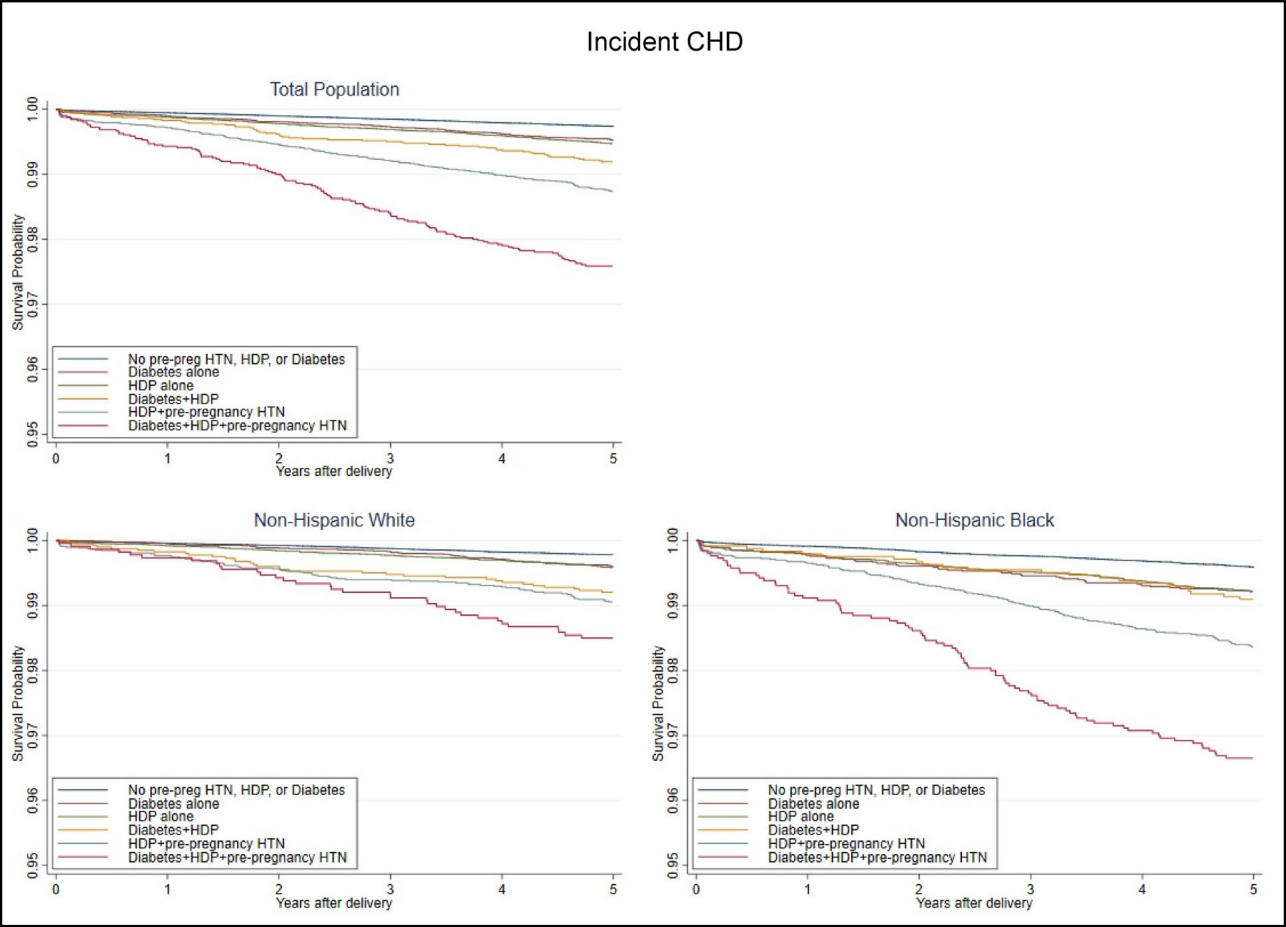
Maternal incident CHD survival within 5 years of delivery by population^a^ and exposure. CHD, coronary heart disease; HDP, hypertensive disorders of pregnancy; HTN, hypertension (pre-pregnancy). ^a^Total population includes Non-Hispanic White, Non-Hispanic Black, and Hispanic women. Hispanic-specific analysis was not run due to low counts

Kaplan-Meier curves for maternal incident CHD within 5 years of delivery are presented with the y-axis truncated in Fig. 3. Incident CHD survival varied by exposure group for the total cohort and among NHB and NHW women, with survival lowest for those with all three conditions. There was no significant difference between women with diabetes only and women with HDP only. However, when comparing women with diabetes only to women with diabetes and HDP or to women with pre-pregnancy HTN, the groups were significantly different.

Model results for the risk of maternal incident CHD within 5 years of delivery and the entire study period are presented overall in Table 2 as there was not a significant interaction with racial/ethnic group. Model 1 adjusted for sociodemographic characteristics, model 2 further adjusted for behavioral characteristics, and model 3 further adjusted for clinical characteristics. After full adjustment, the risk of incident CHD within 5 years of delivery remained elevated for women with diabetes (HR = 1.57, 95% CI 1.28–1.92), HDP (HR = 1.85, 95% CI 1.60–2.15), HDP and diabetes (HR = 2.29, 95% CI 1.73–3.03), HDP and pre-pregnancy HTN (HR = 3.13, 95% CI 2.66–3.68), and all three conditions (HR = 4.87, 95% CI 3.95–6.01) compared to women with no conditions.

**Table 2:** Adjusted ^a^ HRs comparing pre-pregnancy HTN, HDP, and diabetic status for maternal incident CHD

	CHD ≤ 5 years		CHD all follow-up (≤ 14 years)	
	HR (95% CI)		HR (95% CI)	
Model 1				
None	Referent		Referent	
Diabetes	1.63	(1.33–1.99)	1.79	(1.58–2.03)
HDP	1.90	(1.64–2.20)	1.79	(1.62–1.97)
HDP & Diabetes	2.42	(1.83–3.19)	2.86	(2.42–3.38)
HDP & Pre-pregnancy HTN	3.31	(2.82–3.88)	3.21	(2.89–3.56)
HDP, Diabetes, & Pre-pregnancy HTN	5.23	(4.27–6.42)	5.24	(4.57-6.00)
Model 2				
None	Referent		Referent	
Diabetes	1.63	(1.33–1.99)	1.79	(1.58–2.03)
HDP	1.90	(1.65–2.21)	1.79	(1.62–1.97)
HDP & Diabetes	2.43	(1.84–3.21)	2.87	(2.43–3.40)
HDP & Pre-pregnancy HTN	3.31	(2.82–3.88)	3.21	(2.89–3.56)
HDP, Diabetes, & Pre-pregnancy HTN	5.26	(4.29–6.45)	5.28	(4.61–6.06)
Model 3				
None	Referent		Referent	
Diabetes	1.57	(1.28–1.92)	1.65	(1.46–1.88)
HDP	1.85	(1.60–2.15)	1.68	(1.53–1.86)
HDP & Diabetes	2.29	(1.73–3.03)	2.55	(2.15–3.01)
HDP & Pre-pregnancy HTN	3.13	(2.66–3.68)	2.87	(2.58–3.19)
HDP, Diabetes, & Pre-pregnancy HTN	4.87	(3.95–6.01)	4.51	(3.92–5.18)

CHD, coronary heart disease; CI, confidence interval; HDP, hypertensive disorders of pregnancy; HR, hazard ratio; HTN, hypertension

^a^ Model 1 adjusted for sociodemographic characteristics (maternal age, race/ethnicity, education, rural/urban residence, insurance during pregnancy, and WIC (Women, Infants, and Children) receipt during pregnancy

Model 2 adjusted for sociodemographic and behavioral characteristics (smoking during or pre-pregnancy)

Model 3 adjusted for sociodemographic, behavioral, and clinical characteristics (pre-pregnancy BMI, number of births prior to index birth)

Within 14 years of delivery, the risk of incident CHD remained elevated for women with diabetes (HR = 1.65; 95% CI 1.46–1.88), HDP (HR = 1.68; 95% CI 1.53–1.86), HDP and diabetes (HR = 2.55; 95% CI 2.15–3.01), HDP and pre-pregnancy HTN (HR = 2.87; HR = 2.58–3.19), and all three conditions (HR = 4.51; 95% CI 3.92–5.18) compared to those with no conditions after full adjustment.

### Pre-pregnancy HTN, HDP, and/or diabetes with all-cause mortality within 5 years and up to 14 years (entire study period) after delivery

**Fig. 4:**
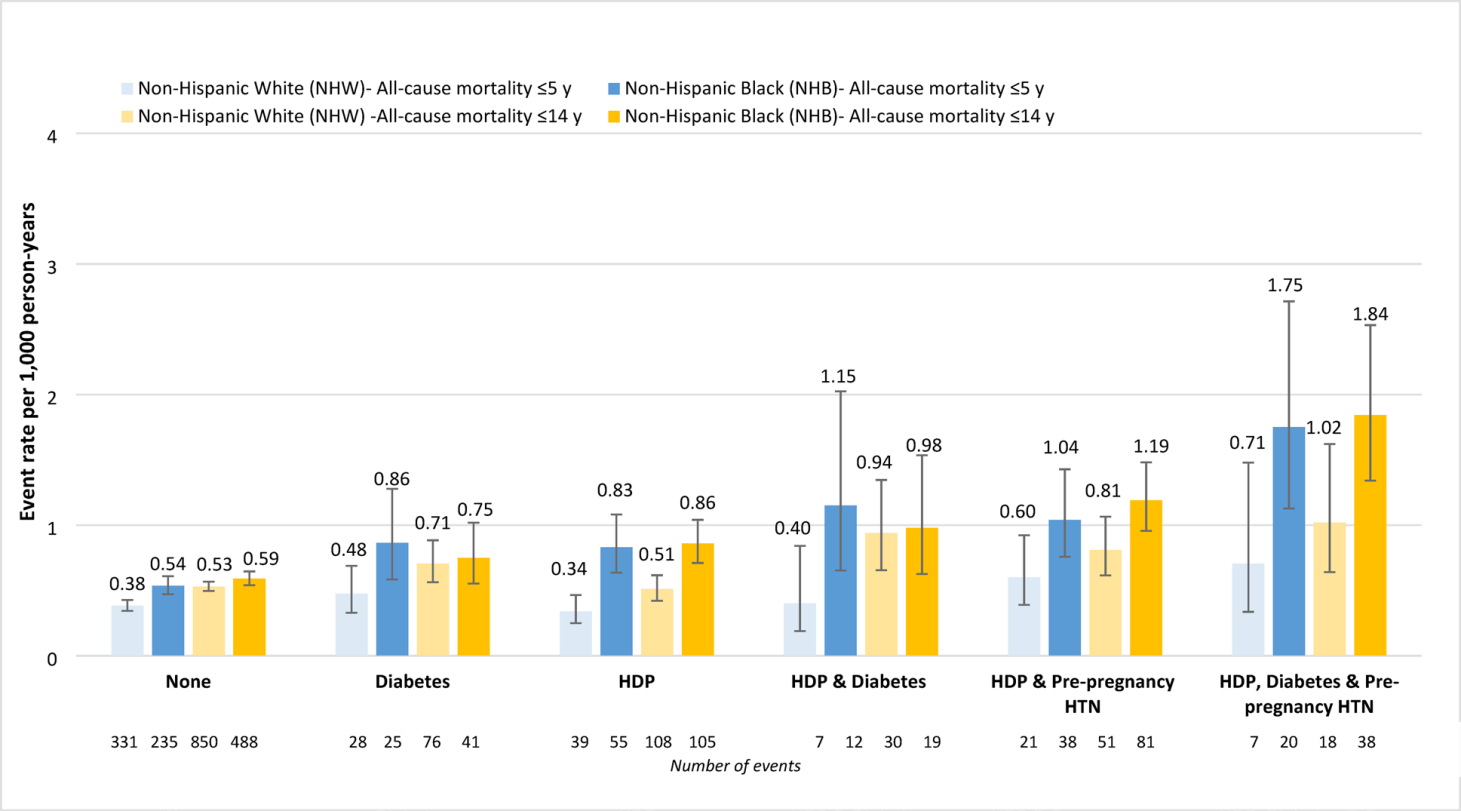
All-cause mortality events (per 1,000 person-years) among women ≤ 5 years following delivery and over the entire study period (≤ 14 years) by race/ethnicity and exposure group ^a^. CI, confidence interval; HDP, hypertensive disorders of pregnancy; HR, hazard ratio; HTN, hypertension (pre-pregnancy); NHB, non-Hispanic Black; NHW, non-Hispanic White. ^a^ The total population included Hispanic women; however, the number of events were too low to present race/ethnic-specific events for Hispanic women

All-cause mortality rates (per 1,000 person-years) within 5 years of delivery were highest among women with all three conditions (1.21, *n* = 27) followed by HDP and pre-pregnancy HTN (0.80, *n* = 59), HDP and diabetes (0.63, *n* = 19), HDP (0.50, *n* = 96), diabetes (0.56, *n* = 55), and no exposure (0.41, *n* = 594) (Supplemental Table 3). As for incident CHD, all-cause mortality rates within 5 years of delivery were highest for NHB women in each exposure group (Fig. 4).

All-cause mortality rates (per 1,000 person-years) within 14 years of delivery were highest among women with all three conditions (1.40, *n* = 56) followed by HDP and pre-pregnancy HTN (0.99, *n* = 134), HDP and diabetes (0.89, *n* = 50), diabetes (0.66, *n* = 120), HDP (0.61, *n* = 216), and no exposure (0.51 per 1,000 person-years, *n* = 1383) (Supplemental Table 3). As shown in Fig. 4, All-cause mortality rates within 14 years of delivery were highest for NHB women in each exposure group, although only slightly higher for those with HDP and diabetes or diabetes alone than NHW women.

**Fig. 5:**
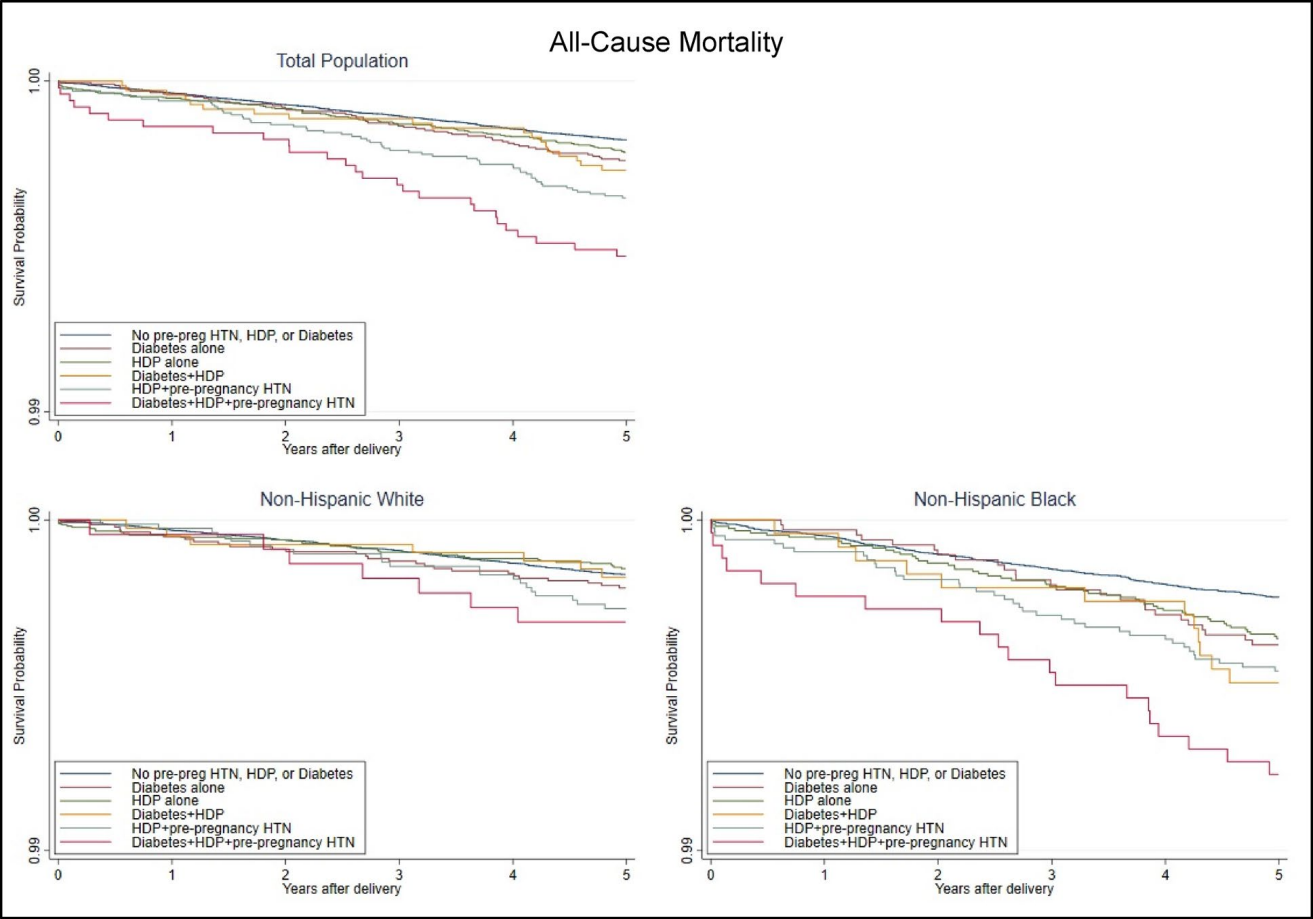
Maternal all-cause mortality within 5 years of delivery by population^a^ and exposure. HDP, hypertensive disorders of pregnancy; HTN, hypertension (pre-pregnancy). ^a^Total population includes Non-Hispanic White, Non-Hispanic Black, and Hispanic women. Hispanic-specific analysis was not run due to low counts

Concerning all-cause mortality, women with all three conditions had the lowest survival rates (Fig. 5). Survival was also lowest for NHB women with all three conditions, whereas for NHW women, all-cause mortality survival appeared similar during the first two years after delivery, declining the most for women with all three conditions. There was no significant difference between women with diabetes only and women with HDP only or women with diabetes and HDP. However, there were significant differences when comparing women with diabetes only to women with pre-pregnancy HTN and HDP.

No significant interaction with race/ethnicity was found in the models built for the risk of maternal all-cause mortality within 5 years after delivery (Table 3). After full adjustment, all-cause mortality risk within 5 years remained elevated for women with diabetes (HR = 1.34; 95% CI 1.01–1.78), HDP and pre-pregnancy HTN (HR = 1.53; 95% CI 1.15–2.03), and all three conditions (HR = 2.25; 95% CI 1.51–3.36) compared to none, although was not significantly elevated for those with HDP or both HDP and diabetes.

**Table 3:** Adjusted* HRs comparing pre-pregnancy HTN, HDP, and diabetic status for maternal all-cause mortality

	All-cause mortality ≤ 5 years		All-cause mortality all follow-up (≤ 14 years)	
	HR (95% CI)		HR (95% CI)	
Model 1				
None	Referent		Referent	
Diabetes	1.31	(0.99–1.74)	1.23	(1.02–1.49)
HDP	1.14	(0.92–1.42)	1.12	(0.96–1.29)
HDP & Diabetes	1.35	(0.86–2.14)	1.60	(0.96–1.29)
HDP & Pre-pregnancy HTN	1.50	(1.13–1.98)	1.57	(1.30–1.88)
HDP, Diabetes, & Pre-pregnancy HTN	2.17	(1.46–3.22)	2.09	(1.59–2.75)
Model 2				
None	Referent		Referent	
Diabetes	1.31	(0.99–1.74)	1.24	(1.02–1.50)
HDP	1.15	(0.93–1.43)	1.13	(0.97–1.30)
HDP & Diabetes	1.36	(0.86–2.16)	1.61	(1.21–2.14)
HDP & Pre-pregnancy HTN	1.50	(1.13–1.98)	1.56	(1.30–1.88)
HDP, Diabetes, & Pre-pregnancy HTN	2.19	(1.48–3.26)	2.12	(1.61–2.79)
Model 3				
None	Referent		Referent	
Diabetes	1.34	(1.01–1.78)	1.25	(1.03–1.51)
HDP	1.17	(0.94–1.46)	1.13	(0.98–1.31)
HDP & Diabetes	1.40	(0.88–2.22)	1.62	(1.22–2.15)
HDP & Pre-pregnancy HTN	1.53	(1.15–2.03)	1.56	(1.30–1.89)
HDP, Diabetes, & Pre-pregnancy HTN	2.25	(1.51–3.36)	2.12	(1.61–2.81)

CI, confidence interval; HDP, hypertensive disorders of pregnancy; HR, hazard ratio; HTN, hypertension

^a^ Model 1 adjusted for sociodemographic characteristics (maternal age, race/ethnicity, education, rural/urban, income, payer, and WIC [Women, Infants, and Children] receipt during pregnancy)

Model 2 adjusted for sociodemographic and behavioral characteristics (smoking during or pre-pregnancy)

Model 3 adjusted for sociodemographic, behavioral, and clinical characteristics (pre-pregnancy BMI, number of births prior to index)

Within 14 years of delivery, all-cause mortality risk was increased for women with diabetes (HR = 1.25; 95% CI 1.03–1.51), HDP and diabetes (HR = 1.62; 95% CI 1.22–2.15), HDP and pre-pregnancy HTN (HR = 1.56; HR = 1.30–1.89), and all three conditions (HR = 2.12; 95% CI 1.61–2.81) after full adjustment compared to those with no conditions (Table 3). The risk of all-cause mortality within 14 years of delivery was not statistically increased for women with HDP.

## Discussion

Subsequent maternal incident CHD and all-cause mortality event rates were highest overall for women who experienced three conditions (HDP, diabetes [during or prior to pregnancy], and pre-pregnancy HTN) compared to women with none of the conditions. Women with two conditions (HDP and diabetes, or HDP and pre-pregnancy HTN) were also more likely to have an incident CHD event or all-cause mortality than women with none of the conditions. As the number of conditions increased, the incidence of CHD and all-cause mortality increased. Furthermore, all rates of subsequent maternal incident CHD events and all-cause mortality were higher in NHB than NHW women regardless of the exposure category.

All of this suggests that HDP has an impact on incident CHD events and all-cause mortality, and that the impact varies with additional comorbidities. Our results are relatively consistent with findings from other studies when considering single or double exposure associations; however, evidence is lacking regarding the joint effect of HDP, pre-pregnancy HTN, and diabetes. We had the ability to adjust for individual-level covariates including sociodemographic, behavioral, and clinical characteristics and to examine potential differences by racial/ethnic group. Pre-pregnancy BMI was included in the final model as it is an independent risk factor for both CHD and all-cause mortality, consistent with the literature.[24, 25] As BMI is on the causal pathway for the development of diabetes [26], it was not included in models 1 and 2.

### CHD hazard

With regard to CHD risk, we found that the hazard increased significantly in women with HDP albeit differently depending on additional comorbidities. HRs ranged from 1.85 in women with HDP alone to 4.51 in women with HDP, diabetes, and pre-pregnancy hypertension. This finding is similar to that seen in a 2022 meta-analysis of 10 studies including 5,168,215 women in which the risk of ischemic heart disease was 2-fold higher (relative risk = 2.06, 95% CI 1.38–3.08) for past HDP than normotensive pregnancies [27]. 

We found that women with HDP and/or diabetes exhibited HRs ranging from 1.57 to 2.87, similar to the risk of incident CVD seen in a 2022 Canadian cohort study of 886,295 women with 12 years of follow-up among women with and without gestational HTN and/or GDM [28]. Within 5 years of delivery, they reported a 1.9-fold (95% CI 1.51–2.35) elevation in incident CVD risk for women with gestational HTN compared to neither condition after controlling for relevant cardiometabolic risk factors [28]. More than 5 years after delivery, there was a 2.4-fold (95% CI 1.60–3.67) increased risk of incident CVD among women with both gestational hypertension and GDM compared to neither condition [28]. 

Many prior studies have focused on either GDM or HDP rather than the joint impact of both on incident CHD and all-cause mortality. In a 2021 review, the association between type of HDP and myocardial infection ranged from an odds ratio (OR) of 3.0 (95% CI 2.0-4.6) for preeclampsia to an OR of 5.2 (95% CI 3.1–8.7) for preeclampsia superimposed on chronic HTN, which corresponds to our finding of an increased risk of CHD in women with HDP and/or pre-pregnancy hypertension [11]. Another study found the odds of cardiovascular-related morbidity increased for women with gestational HTN (OR = 1.67, 95% CI 1.28–2.19) and moderate (OR = 2.24, 95% CI 1.72–2.93) or severe preeclampsia (OR = 2.74, 95% CI 2.48–3.04) compared to those without the conditions [13]. Within the same cohort as the current study, we previously reported elevated incident CHD in women with HDP and pre-pregnancy HTN (HR = 3.79; 95% CI 3.09–4.65), HDP (HR = 2.32; 95% CI 2.03–2.65), and pre-pregnancy HTN (HR = 2.48; 95% CI 1.40–4.40) following adjustment; our current findings are similar if slightly attenuated [12]. Similar to our findings in women with diabetes, a meta-analysis reported an association between GDM (OR = 1.68; 95% CI 1.11–2.52) and CVD-related morbidity and mortality [13]. Another meta-analysis of 9 studies involving 5,390,591 women from 2019 reported twice the risk of future CVD events among women with GDM compared to those without (RR = 1.98, 95% CI 1.57–2.50) [29]. The association attenuated although remained significant when limited to women who did not develop type 2 diabetes (RR = 1.56, 95% CI 1.04–2.32) [29]. Within 10 years after delivery, the risk of future cardiovascular events was even higher among studies with ≤ 10 years follow-up of GDM (RR = 2.31, 95% CI 1.57–3.39) [29]. More recently, a meta-analysis of 38 studies involving 77,678,684 participants reported an association between GDM and overall CVDs post-pregnancy (risk ratio = 1.46, 95% CI 1.34–1.59) [30]. In contrast to these prior studies, we examined the combined exposure of having HDP, pre-pregnancy HTN, and diabetes on incident CHD risk within 5 years of delivery and report an elevated HR of 4.87 (95% CI 3.95–6.01).

## All-cause mortality hazard

As with CHD risk, the joint effect of diabetes and HDP on all-cause mortality has not been studied in depth; the majority of studies have focused on the association of HDP with all-cause mortality. Our reported risk of mortality within 5 years after delivery (ranging from a HR of 1.17 in women with HDP alone to a HR of 2.25 in women with all three conditions) was similar to that reported in a review where the adjusted odds for the relationships between chronic HTN and preeclampsia with maternal mortality ranged from 1.7 (95% CI 1.2–2.4) to 2.6 (95% CI 2.1–3.4), respectively [11], as well as a study that reported the association of preeclampsia with elevated odds of cardiovascular-related mortality (OR = 1.73, 95% CI 1.46–2.06) [13]. We previously reported increased all-cause mortality risk within 5 years of delivery among women with both HDP and pre-pregnancy HTN (HR = 2.21; 95% CI 1.61–3.03) and HDP alone (HR = 1.35; 95% CI 1.13–1.60) following adjustment [12]. Similar to CHD risk, current findings for all-cause mortality risk within 5 years of delivery in women with both HDP and pre-pregnancy HTN (HR = 1.53, 95% CI = 1.15–2.03) and in women with HDP alone (HR = 1.17, 95% CI = 0.94–1.46) are increased although attenuated.

## Limitations

There are some limitations of this investigation. First, due to the data collection methods, pre-pregnancy diabetes and GDM could not be considered separately. With regard to the validation of GDM, a 2023 study evaluated the accuracy of GDM diagnosis between 1998 and 2016 based upon ICD codes and birth certificates in a large hospital-based cohort of pregnant women (*n* = 51,059) [31]. A total of 1303 women (2.6%) met the laboratory criteria for GDM, for which the specificity (99.3%, 95% CI 99.3–99.4) of ICD codes was high, whereas the sensitivity (70.5%, 95% CI 67.9–72.9) was moderate [31]. Among pregnancies linked to birth certificate data (*n* = 46,512), birth certificate diagnosis of GDM similarly had high specificity (98.9%, 95%: CI 98.8–99.0), whereas the sensitivity (66.3%, 95% CI 63.6–69.0) was moderate [31]. Results from this validation study indicate that while reported GDM was likely a valid case, roughly 305 of true GDM cases were likely missed by relying on administrative data. Second, low counts in this dataset led to the exclusion of two exposure groups (women with pre-pregnancy HTN alone, and women with both pre-pregnancy HTN and diabetes) and races/ethnicities other than NHW, NHB, and Hispanic women. Even with the inclusion of these groups, the interactions between the exposures and race/ethnicity were not significant, and the number of events for both incident CHD and all-cause mortality were too low among Hispanic women for analysis. If the data analyzed covered a longer time period, this could increase the likelihood of events and strengthen the findings. Third, pregnancies occurring after the index pregnancy were not addressed in this analysis. We adjusted for parity at the time of the index pregnancy but did not adjust for births after the index birth because it could be viewed as overcontrolling. This adjustment is consistent with other literature [32]. As information on post-partum treatment was not available in the data sources, and specifically antihypertensive treatment and insulin use, we were unable to adjust for them.

## Conclusions

In summary, incident CHD and all-cause mortality rates were highest for women with two or three conditions (HDP and diabetes; HDP and pre-pregnancy HTN; HDP, diabetes, pre-pregnancy HTN), with all rates higher for NHB than NHW women within each of the exposure categories. Within 5 years of delivery, the risk of incident CHD risk was increased and particularly among women with two or three conditions compared to women without any condition after adjustment. Findings were also increased for women with all-cause mortality especially those with two or three of the above conditions except for both HDP and diabetes. This investigation was limited to maternal outcomes within 5 years following delivery and the entire study period of up to 14 years after delivery. Extending the follow-up time may provide more power for the results discovered here and allow for analysis of potential racial/ethnic differences. Such data can help design strategies to improve risk factor screening and identification among all women of child-bearing age. Our findings highlight the importance of implementing clinical practice strategies that could include patient education, cardiovascular risk factor modification, early identification, and adequate post-partum follow-up care. As these women are at higher risk, they should be screened and treated to guidelines if CVD risk factors are found during or after pregnancy.

## Electronic supplementary material

Below is the link to the electronic supplementary material.

Supplementary Material 1

## Data Availability

The data used for this study cannot be shared due to policies of the South Carolina Revenue and Fiscal Affairs Office, Health and Demographics Section and the South Carolina Department of Health and Environmental Control.

## Abbreviations

ANOVA: Analysis of variance
BMI: Body mass index
CDC: Centers for Disease Control and Prevention
CHD: Coronary heart disease
CI: Confidence interval
CVD: Cardiovascular disease
ED: Emergency department
GDM: Gestational diabetes mellitus
HDP: Hypertensive disorders of pregnancy
HR: Hazard ratio
HTN: Hypertension
ICD-9-CM: International Classification of Diseases, Ninth Revision, Clinical Modification
ICD-10-CM: International Classification of Diseases, Tenth Revision, Clinical Modification
NCHS: National Center for Health Statistics
NHB: Non-Hispanic Black
NHW: Non-Hispanic White
OR: Odds ratio
RUCA: Rural-Urban Commuting Area
SC: South Carolina
U.S.: United States
WIC: Special Supplemental Nutrition Program for Women, Infants, and Children
