# Supplementary material for: Maternal coronary heart disease and mortality following hypertensive disorders of pregnancy and/or diabetes
Source: Cardiovasc Diabetol. 2025 Jul 11;24:282. doi: 10.1186/s12933-025-02811-8 (PMC12247428; doi:10.1186/s12933-025-02811-8)
Supplement: Supplementary file 1 — Supplementary Material 1 [file 12933_2025_2811_MOESM1_ESM.docx]

**Supplemental Table 1.** Definitions for the Exposures and Outcomes of Interest

|  | **Source** | **ICD-9-CM code** | **ICD-10-CM code** |
| --- | --- | --- | --- |
| **Pre-existing (pre-pregnancy) hypertension** | Birth certificate, inpatient hospitalization/ED visit encounter data | 642.0-642.2, 642.9 | O10, O11 |
| **Hypertensive disorders of pregnancy (HDP)** |  |  |  |
| Hypertension; complicating pregnancy | Inpatient hospitalization/ED visit encounter data | 642.0-642.2, 642.9 | O10 |
| Eclampsia | Inpatient hospitalization/ED visit encounter data | 642.6 | O15 |
| Pre-existing hypertension with pre-eclampsia | inpatient hospitalization/ED visit encounter data | 642.7 | O12 |
| Pre-eclampsia | inpatient hospitalization/ED visit encounter data | 642.4-642.5, 642.7 | O11, O12, O14 |
| Gestational hypertension | Birth certificate, inpatient hospitalization/ED visit encounter data | 642.3 | O13, O16 |
| **Diabetes** |  |  |  |
| Gestational diabetes | Inpatient hospitalization/ED visit encounter data | 648.8 | O24.4, O24.9 |
| Pre-pregnancy diabetes | Birth certificate, inpatient hospitalization/ED visit encounter data | 250.00-250.92, 648.0 (maternal) | E10.x, E11.x, O24.3 |
| **Coronary heart disease (CHD)** | Inpatient hospitalization/ED visit encounter data | 410.x-414.x | I20.x, I21.x I24.x, I25.x |
| Myocardial infarction | Inpatient hospitalization/ED visit encounter data | 410.x | I21.x |
| Cardiovascular disease, unspecified | Inpatient hospitalization/ED visit encounter data | 429 | I25.10 |
| Atherosclerosis | Inpatient hospitalization/ED visit encounter data | 440 | I70 |
| Cardiomegaly | Inpatient hospitalization/ED visit encounter data | 429.3 | I42 |
| **All-cause mortality** | Death certificate  (ICD-10 codes) | -- | -- |

Abbreviations: BC, birth certification; ED, emergency department; ICD-9-CM, International Classification of Diseases, Ninth Revision, Clinical Modification; ICD-10-CM, International Classification of Diseases, Tenth Revision, Clinical Modification

**Supplemental Table 2.** Underlying Cause of Death Listed Based on ICD-10 codes

| Underlying cause of death | N | % ^a^ |
| --- | --- | --- |
| Injury, poisoning and certain other consequences of external causes | 1029 | 46.5 |
| Neoplasms | 350 | 15.8 |
| Diseases of the circulatory system | 315 | 14.2 |
| Pregnancy, childbirth and the puerperium | 138 | 6.2 |
| Diseases of the respiratory system | 70 | 3.2 |
| Endocrine, nutritional and metabolic diseases | 64 | 2.9 |
| Diseases of the digestive system | 56 | 2.5 |
| Certain infectious and parasitic diseases | 55 | 2.5 |
| Diseases of the nervous system | 46 | 2.1 |
| Symptoms, signs and abnormal clinical and laboratory findings, not elsewhere classified | 28 | 1.3 |
| Diseases of the musculoskeletal system and connective tissue | 20 | 0.9 |
| Diseases of the genitourinary system | 16 | 0.7 |
| Mental, Behavioral and Neurodevelopmental disorders | 15 | 0.7 |
| Congenital malformations, deformations and chromosomal abnormalities | 7 | 0.3 |
| Diseases of the skin and subcutaneous tissue | 2 | 0.1 |
| Total | 2211 | 100 |

**Supplemental Table 3.** Event rate per 1,000 comparing pre-pregnancy HTN, HDP, and diabetic status for maternal incident CHD and all-cause mortality within 5 years of delivery and the entire study period (≤14 years) ^a^

|  |  | **Total** | | **NHW** | | | **NHB** | | | **Hispanic** | | |
| --- | --- | --- | --- | --- | --- | --- | --- | --- | --- | --- | --- | --- |
|  | **events** | **event rate** | **(95% CI)** | **events** | **event rate** | **(95% CI)** | **events** | **event rate** | **(95% CI)** | **events** | **event rate** | **(95% CI)** |
| **CHD, ≤5 years** |  |  |  |  |  |  |  |  |  |  |  |  |
| None | 874 | 0.61 | (0.57-0.65) | 439 | 0.51 | (0.46-0.56) | 408 | 0.93 | (0.84-1.03) | 27 | 0.19 | (0.13-0.28) |
| Diabetes | 110 | 1.11 | (0.92-1.34) | 55 | 0.94 | (0.72-1.22) | 52 | 1.80 | (1.37-2.36) | 3 | 0.27 | (0.09-0.83) |
| HDP | 235 | 1.22 | (1.08-1.39) | 110 | 0.95 | (0.79-1.15) | 119 | 1.81 | (1.51-2.16) | 6 | 0.54 | (0.24-1.21) |
| HDP & Diabetes | 57 | 1.88 | (1.45-2.44) | 32 | 1.85 | (1.31-2.61) | 22 | 2.11 | (1.39-3.20) | <5 | 1.19 | (0.38-3.69) |
| HDP & Pre-pregnancy HTN | 211 | 2.87 | (2.51-3.28) | 74 | 2.14 | (1.70-2.68) | 136 | 3.73 | (3.15-4.41) | <5 | 0.41 | (0.06-2.90) |
| HDP, Diabetes, & Pre-pregnancy HTN | 123 | 5.55 | (4.65-6.62) | 34 | 3.44 | (2.46-4.82) | 87 | 7.72 | (6.26-9.53) | <5 | 1.97 | (0.49-7.86) |
| **CHD, all follow-up** |  |  |  |  |  |  |  |  |  |  |  |  |
| None | 2138 | 0.79 | (0.76-0.83) | 1059 | 0.66 | (0.62-0.70) | 1009 | 1.22 | (1.15-1.30) | 70 | 0.26 | (0.21-0.33) |
| Diabetes | 295 | 1.62 | (1.45-1.82) | 150 | 1.40 | (1.19-1.64) | 134 | 2.47 | (2.09-2.93) | 11 | 0.55 | (0.30-0.99) |
| HDP | 529 | 1.50 | (1.38-1.64) | 244 | 1.16 | (1.03-1.32) | 276 | 2.27 | (2.02-2.55) | 9 | 0.44 | (0.23-0.85) |
| HDP & Diabetes | 156 | 2.81 | (2.40-3.29) | 77 | 2.44 | (1.95-3.05) | 74 | 3.83 | (3.05-4.81) | 5 | 1.08 | (0.45-2.59) |
| HDP & Pre-pregnancy HTN | 477 | 3.56 | (3.26-3.90) | 160 | 2.56 | (2.19-2.98) | 310 | 4.63 | (4.14-5.17) | 7 | 1.61 | (0.77-3.37) |
| HDP, Diabetes, & Pre-pregnancy HTN | 264 | 6.75 | (5.99-7.62) | 81 | 4.68 | (3.76-5.82) | 179 | 8.93 | (7.71-10.33) | <5 | 2.33 | (0.87-6.20) |
| **All-cause mortality, ≤5 years** |  |  |  |  |  |  |  |  |  |  |  |  |
| None | 594 | 0.41 | (0.38-0.45) | 331 | 0.38 | (0.34-0.43) | 235 | 0.54 | (0.47-0.61) | 28 | 0.20 | (0.14-0.29) |
| Diabetes | 55 | 0.56 | (0.43-0.72) | 28 | 0.48 | (0.33-0.69) | 25 | 0.86 | (0.58-1.28) | <5 | 0.18 | (0.04-0.72) |
| HDP | 96 | 0.50 | (0.41-0.61) | 39 | 0.34 | (0.25-0.46) | 55 | 0.83 | (0.64-1.09) | <5 | 0.18 | (0.05-0.72) |
| HDP & Diabetes | 19 | 0.63 | (0.40-0.98) | 7 | 0.40 | (0.19-0.84) | 12 | 1.15 | (0.65-2.02) | 0 | 0.00 | -- |
| HDP & Pre-pregnancy HTN | 59 | 0.80 | (0.62-1.03) | 21 | 0.60 | (0.39-0.93) | 38 | 1.04 | (0.75-1.42) | 0 | 0.00 | -- |
| HDP, Diabetes, & Pre-pregnancy HTN | 27 | 1.21 | (0.83-1.76) | 7 | 0.71 | (0.34-1.48) | 20 | 1.75 | (1.13-2.72) | 0 | 0.00 |  |
| **All-cause mortality, all follow-up** |  |  |  |  |  |  |  |  |  |  |  |  |
| None | 1383 | 0.51 | (0.49-0.54) | 850 | 0.53 | (0.50-0.57) | 488 | 0.59 | (0.54-0.65) | 45 | 0.17 | (0.13-0.23) |
| Diabetes | 120 | 0.66 | (0.55-0.79) | 76 | 0.71 | (0.56-0.88) | 41 | 0.75 | (0.55-1.02) | <5 | 0.15 | (0.05-0.46) |
| HDP | 216 | 0.61 | (0.54-0.70) | 108 | 0.51 | (0.42-0.62) | 105 | 0.86 | (0.71-1.04) | <5 | 0.15 | (0.05-0.46) |
| HDP & Diabetes | 50 | 0.89 | (0.68-1.18) | 30 | 0.94 | (0.66-1.35) | 19 | 0.98 | (0.62-1.53) | <5 | 0.21 | (0.03-1.52) |
| HDP & Pre-pregnancy HTN | 134 | 0.99 | (0.84-1.17) | 51 | 0.81 | (0.61-1.06) | 81 | 1.19 | (0.96-1.48) | <5 | 0.46 | (0.11-1.83) |
| HDP, Diabetes, & Pre-pregnancy HTN | 56 | 1.40 | (1.08-1.82) | 18 | 1.02 | (0.65-1.63) | 38 | 1.84 | (1.34-2.53) | 0 | 0.00 | -- |

Abbreviations: CHD, coronary heart disease; CI, confidence interval; HDP, hypertensive disorders of pregnancy; HTN, hypertension; NHB, non-Hispanic Black; NHW, non-Hispanic White
